# Limits to the scope of non-invasive prenatal testing (NIPT): an analysis of the international ethical framework for prenatal screening and an interview study with Dutch professionals

**DOI:** 10.1186/s12884-018-2050-4

**Published:** 2018-10-19

**Authors:** A. Kater-Kuipers, E. M. Bunnik, I. D. de Beaufort, R. J. H. Galjaard

**Affiliations:** 1 Department of Medical Ethics and Philosophy of Medicine, Erasmus MC, University Medical Centre Rotterdam, Room 24.17, Wytemaweg 80, 3015 CN Rotterdam, The Netherlands; 2 Department of Clinical Genetics, Erasmus MC, University Medical Centre Rotterdam, Wytemaweg 80, 3015 CN Rotterdam, The Netherlands

**Keywords:** Prenatal screening, Non-invasive prenatal testing (NIPT), Reproductive autonomy, Informed consent, Public health ethics, Ethical framework, Qualitative interview study

## Abstract

**Background:**

The introduction of non-invasive prenatal testing (NIPT) for foetal aneuploidies is currently changing the field of prenatal screening in many countries. As it is non-invasive, safe and accurate, this technique allows for a broad implementation of first-trimester prenatal screening, which raises ethical issues, related, for instance, to informed choice and adverse societal consequences. This article offers an account of a leading international ethical framework for prenatal screening, examines how this framework is used by professionals working in the field of NIPT, and presents ethical guidance for the expansion of the scope of prenatal screening in practice.

**Methods:**

A comparative analysis of authoritative documents is combined with 15 semi-structured interviews with professionals in the field of prenatal screening in the Netherlands. Data were recorded, transcribed verbatim and analysed using thematic analysis.

**Results:**

The current ethical framework consists of four pillars: the aim of screening, the proportionality of the test, justice, and societal aspects. Respondents recognised and supported this framework in practice, but expressed some concerns. Professionals felt that pregnant women do not always make informed choices, while this is seen as central to reproductive autonomy (the aim of screening), and that pre-test counselling practices stand in need of improvement. Respondents believed that the benefits of NIPT, and of an expansion of its scope, outweigh the harms (proportionality), which are thought to be acceptable. They felt that the out-of-pocket financial contribution currently required by pregnant women constitutes a barrier to access to NIPT, which disproportionally affects those of a lower socioeconomic status (justice). Finally, professionals recognised but did not share concerns about a rising pressure to test or discrimination of disabled persons (societal aspects).

**Conclusions:**

Four types of limits to the scope of NIPT are proposed: NIPT should generate only test outcomes that are relevant to reproductive decision-making, informed choice should be (made) possible through adequate pre-test counselling, the rights of future children should be respected, and equal access should be guaranteed. Although the focus of the interview study is on the Dutch healthcare setting, insights and conclusions can be applied internationally and to other healthcare systems.

## Background

Non-invasive prenatal testing (NIPT) is based on the analysis of cell free foetal DNA for chromosomal abnormalities [1]. Non-invasiveness refers to the way the foetal DNA sample is obtained: not from the placenta or amniotic fluid, which requires an invasive procedure, but from a blood sample of the mother. NIPT for chromosomal abnormalities was first offered in 2011, in the United States of America, Western-Europe and China [2]. In the Netherlands, prenatal screening for untreatable disorders is subjected to licensing under the Population Screening Act. NIPT has been offered to high-risk women, exclusively within the context of the TRIDENT-1 study (Trial by Dutch laboratories for Evaluation of Non-Invasive Prenatal Testing) since early 2014 [3]. When a woman received a high-risk outcome (chance ≥1:200) from the first trimester combined test (FCT) and wanted further testing, she was offered the choice between NIPT or invasive testing [3]. Low-risk pregnant women who wanted NIPT could not access NIPT in the Netherlands, and went abroad. Since April 2017, NIPT is also offered within the TRIDENT-2 study to low-risk pregnant women, who are given a choice between FCT and NIPT. The current NIPT-based prenatal test includes detection of trisomy 21 (Down syndrome), trisomy 18 (Edwards syndrome) and trisomy 13 (Patau syndrome). Compared to the FCT the sensitivity, the specificity and the positive predictive value of NIPT are remarkably high for those trisomies [3]. The positive predictive value is slightly lower in low-risk pregnant women but still higher than the positive predictive value of the FCT [1, 4, 5]. However, NIPT is not a diagnostic test. This is because of several factors. First, cell free foetal DNA circulating in maternal blood originates from the placenta, not the foetus. The presence of a chromosomal anomaly can be limited to the placenta in case of confined placental mosaicism without affecting the foetus, thus resulting in a false positive NIPT outcome. Furthermore, the presence of maternal chromosomal anomalies, including those originating from a maternal tumour, low foetal DNA fraction in maternal blood, and a vanishing twin [6, 7], may still lead to inconclusive, false positive or false negative results [8]. Yet NIPT leads to fewer unnecessary invasive follow-ups tests - through amniocentesis and chorionic villus sampling - than the FCT. These invasive tests have a 0.1–0.2% risk, respectively, of miscarriage [3]. To minimize the need for invasive testing – and the associated risk of miscarriage – has been one of the major reasons for implementing NIPT in current screening programmes.

When genome-wide sequencing techniques are used to perform NIPT, they allow for the detection of chromosomal abnormalities other than trisomy 13, 18 and 21, and thus for an expansion of the current scope of prenatal screening [3, 9]. Several studies have indicated that additional findings could include other full trisomies, sex-chromosomal abnormalities, and sub-chromosomal aberrations, associated with rare diseases [3, 10–12]. However, the possibility to find more abnormalities has raised questions, notably the policy question whether the screening offer should be expanded to include all those abnormalities. When discussing the question whether or not to include additional conditions, experts have brought up considerations of clinical utility and concerns related to the consequences of a broader test for informed choice [13–15]. These concerns were raised 10 years ago, when genome-wide arrays were introduced in prenatal diagnosis, and are raised again with renewed urgency in the context of the introduction of NIPT [16].

Various statements and position papers about prenatal screening, issued by governmental organisations and ethical committees, have addressed ethical issues of prenatal screening [15, 17–24]. Together with scholarly studies of ethical issues in prenatal screening [25–30] these statements and position papers could be seen as an – unofficial, but broadly shared and often referred to – international ethical framework for prenatal screening. An ethical framework can be defined as a specification of general principles in a specific context, through which the scope of general principles is narrowed by spelling out why and how actions should be undertaken or avoided [31]. The aim of an ethical framework is to “provide practical guidance for public health professionals and to highlight the defining values of public health” [32].

The aim of this article is firstly to reconstruct and analyse the main tenets of the ethical framework for prenatal screening and then to compare these with the practice of prenatal screening, through interviews with professionals in the field of prenatal screening. Secondly, this article examines whether and how the ethical framework can guide an expansion of the scope of prenatal screening.

## Methods

For this study we used a combination of methods, a literature study and a qualitative interview study. We conducted a comparative analysis of ethical statements about requirements for (non-invasive) prenatal testing formulated by national and international organisations or committees. Also, we conducted in-depth interviews with Dutch professionals in the field of prenatal screening. The interviews serve to illustrate how the ethical framework for prenatal screening is translated into practice, and to offer insight into professionals’ moral views on recent developments in prenatal screening.

### Document analysis

To identify important documents that represent an ethical framework we started with an authoritative article of the European Society of Human Genetics (ESHG) and the American Society of Human Genetics (ASHG), which offers a consensus view of responsible innovation in prenatal screening, which is also endorsed by the Human Genetics Society of Australasia and other related professional associations in Europe [15]. We built on this consensus view and postulated four pillars of an ethical framework: the aim of prenatal screening, proportionality of testing, justice, and societal aspects. Other studies and documents – notably from the World Health Organisation (WHO), UNESCO International Bioethics Committee (IBC), German Ethics Council (Ethikrat) and the Dutch Health Council (GR) – were reviewed to corroborate, adapt and complement the ethical framework. We selected these four other documents for our analysis because – in contrast to other publications we have reviewed – these documents contain discussions of issues related to all four pillars.

### Interviews

For the qualitative interview study, professionals in the field of prenatal screening and follow-up diagnostic testing from six academic centres in the Netherlands were invited. In total 15 individual in-depth interviews were conducted with two midwives, seven medical specialists (three gynaecologists, four clinical geneticists specialised in prenatal diagnosis), two lab specialists working with NIPT, two test developers and two policy makers. The interviews were conducted at the respondents’ work places or at Erasmus MC. A semi-structured interview guide was used. This guide included five themes: informed decision-making, proportionality, access to NIPT, societal aspects and the scope of prenatal screening. Interviews were digitally recorded, transcribed verbatim and analysed with Atlas.ti using thematic analysis, based on the five indicated themes.

## Results

The documents each point at the four pillars - the aim of screening, the proportionality of the test, justice, and societal aspects - but differ in some aspects of their interpretations. Table 1 presents an overview of interpretations of the four pillars in the five documents. Below we present the four pillars of the ethical framework for the practice of prenatal screening, complemented with results from the interviews.

**Table 1:** An ethical framework for prenatal screening

	ESHG/ASHG (2015)	WHO (2003)	Ethikrat (2013)	Dutch Health Council (2013)	UNESCO (2015)
*Aim of prenatal screening*	The aim is to enable autonomous reproductive choices, i.e. meaningful choices, related to serious health problems. The aim is achieved when women are enabled to make informed choices. Prenatal screening has a different goal than other forms of screening, because of the ‘morally sensitive practice’ of (selective) abortion and the stigmatisation of disabled people.	The aim is to obtain information and to promote freedom of choice and autonomy. Being able to prepare for the birth of a child with a disability is also seen as a way to exercise reproductive autonomy. Free choice requires: 1) adequate, unbiased information; 2) availability of relevant alternatives, including availability of healthcare services for disabled children or the (legal) possibility of abortion.	The aim is not specified, but prenatal screening is linked to self-determination and autonomy: “If a pregnant woman makes decisions about her pregnancy, these must be seen inter alia in the context of her right to reproductive self-determination.” Reproductive decisions affect the unborn child and are thus not unlimited.	The aim is to enable and promote choice concerning terminating or continuing the pregnancy. “Informed choice is not a condition for, but the aim of prenatal screening.” The aim is not to maximise reproductive choice as such. If informed choice is not reached, the aim is not achieved.	The aim is “not health gain but to decide (…) whether to carry a pregnancy to term.” Furthermore, “it allows those involved to prepare for the birth of a sick or disabled child.” There is controversy about the limits of reproductive autonomy in the light of a child’s right to an un-manipulated genetic make-up. Prevention, focused on “reducing care costs for people with congenital conditions or disabilities, cannot be the goal of such screening. That would imply a discriminatory practice that sends the message that these people are unwelcome in society.”
Proportionality	Proportionality is defined as a balancing of benefits and harms. Benefits of NIPT include reassurance, assistance in making informed reproductive decisions, and less invasive testing. Harms of prenatal screening generally include false reassurance, stress and anxiety, and risk of miscarriage in follow-up diagnostic testing. Balancing of benefits and harms includes consideration of quality aspects of the test and adequate counselling.	Proportionality is not discussed. Benefits and burdens are included in a cost-benefit analysis. Benefits are the chance to prepare for the birth of a child that will need medical treatment or a relief of maternal anxiety. Burdens include selective abortion of a wanted pregnancy.	Proportionality is not discussed, although it is stated that quality assurance is a precondition to meeting the aim of prenatal screening. Balancing benefits and harms is difficult: “The effect of a differentiated prenatal diagnosis is ambivalent. It may relieve the pregnant woman of fears, but on the other hand there is the danger that the (...) associated burden of deciding make[s] the couple affected (…) and may even overstrain them.” It is seen as a great risk that women may be insufficiently aware of the consequences of testing and subsequent decisions they will have to make.	Proportionality is a central requirement for prenatal screening. Screening is only justified when the value or utility of the offer is established and the benefits outweigh the disadvantages. Benefits of NIPT include reassurance, informed reproductive decisions and less invasive testing. ‘Disadvantages’ of screening include false reassurance, test-related stress and anxiety and risk of miscarriage.	Proportionality and proven usefulness are preconditions for a justified prenatal screening offer. The advantages should outweigh the disadvantages. Benefits are having freedom to choose and fewer invasive tests. Disadvantages are “routinization and institutionalization of choice of not giving birth to an ill or disabled child.” If a test becomes self-evident women might feel pressured to test or stigmatised when they will not test.
Justice	Justice refers to the distribution of costs: “As health budgets are inevitably limited (…), opportunity costs will have to be taken into account as well.” The requirement of just distribution of healthcare costs will demarcate the scope of prenatal screening.	Justice refers to the “equitable distribution of genetics services, including prenatal diagnosis, is owed first to those with the greatest medical need, regardless of ability to pay, or any other considerations.” Relief of maternal anxiety has lower priority than medical indications for prenatal screening, in the just distribution of healthcare resources.	Justice refers to equal access to prenatal screening and to the danger of “discrimination and stigmatization of people with particular genetic characteristics.”	Justice refers to an appropriate use of healthcare resources. Prenatal screening can be used appropriately by couples wishing to prepare for the birth of an affected child, which is considered a justified aim of screening. Justice is also mentioned in the context of equal access to an expanded scope of prenatal screening: screening for abnormalities other than trisomy 21, 18 and 13 should not only be available for high-risk women (through invasive follow-up testing).	Justice refers to the organisation of healthcare systems, so that innovations are shared with society as a whole, without becoming a new source of inequality and discrimination. Justice also pertains to education: people should be actively enabled to exercise their freedom and autonomy. Finally, it refers to proper distribution of costs and investments in various fields within the healthcare system (e.g. care for people with disabilities).
Social aspects	As a public health programme, prenatal screening might have “consequences for other individuals and groups (including those living with the relevant conditions)”. To avoid discriminatory messages, the aim of screening (reproductive autonomy) should be stressed. NIPT might be seen as a routine procedure,which might lead to routinisation of prenatal screening, affecting the informed choices of pregnant women.	Prenatal screening may have negative effects for people with disabilities, but does not lead to the birth of fewer people with disabilities, as long as chromosomal and single gene disorders account for only a minority of disabilities present at birth. Healthcare for people with disabilities will and should not be reduced, also to prevent ‘economic eugenics’ which would hinder voluntary decision-making. Although “cultures or medical settings may be implicitly coercive,” these problems are seen as part of the general sociocultural context and not attributed to prenatal screening specifically.	Prenatal screening generates genetic information and could have social consequences including stigmatisation and discrimination. There is a (shared) duty to create a society without discrimination, which will be a result of interactions between people and does not depend on the presence or absence of one test. Routine offer of prenatal screening might have negative consequences for reproductive freedom and put pressure on women to test: the idea that pregnant women should take their parental responsibility and opt for testing should be avoided.	Prenatal screening programmes may have social impact but are not necessarily discriminatory, because they aim at reproductive choice and not at prevention. However, concerns related to the social impact of screening on people with a disability are realistic. Therefore ongoing ethical monitoring of screening practices is necessary. Also, the state should guarantee good-quality care for people with a disability. A simple and safe test could possibly lead to routinisation, including pressure to test. This might affect reproductive autonomy.	Prenatal screening is often not a therapeutic intervention but likely to lead to abortion and it might lead to discrimination and stigmatisation. “The adding up of a lot of individual choices to the ‘acceptability’ of aborting certain kinds of embryos (…) brings forward a societal phenomenon, which resembles a kind of eugenics in the search for a ‘perfect child’.” Non-discrimination should be emphasised and guaranteed. Prenatal screening as a ‘routine measure’ might negatively affect society’s perception of disability and societal solidarity with disabled people and the women who give birth to them.

### Aim of prenatal screening

The first pillar of the ethical framework for prenatal screening pertains to the aim of prenatal screening for foetal abnormalities. Prenatal screening differs from other areas of public health, where the aim is reduction of morbidity and mortality associated with disorders in the population [15]. Translating this aim to prenatal screening might imply that the success of a prenatal screening programme would be defined in terms of maximisation of the termination rate of foetuses with abnormalities, which would be problematic, as abortion is often a point of controversy [15, 17, 19, 20]. Besides, prenatal screening is thought to imply discriminatory messages about the value of the lives of people living with the relevant conditions [15, 17–19]. The widely supported view therefore is that governments can only justifiably offer prenatal screening when the aim is to enable pregnant women and their partners to make autonomous reproductive choices [15, 17, 19].

Although interviewed professionals recognised informed choice as the aim of prenatal screening, some of them pointed out that prenatal screening also provides the opportunity to prepare for the birth of a disabled child and to improve the care for it. Several respondents thought that the latter should be emphasised more during pre-test counselling and that it should be made clear that prenatal screening is not exclusively aimed at offering women the opportunity to terminate an affected pregnancy.

The right not to know about the options of prenatal screening is considered very important. In the Netherlands, this has been formalised in the obligation of professionals to present women with an ‘information offer’ first [33], in order to stress the fact that prenatal screening for aneuploidies is not mandatory. When a pregnant woman visits the midwife or obstetrician, the professional must first ask whether the woman wants to be informed about prenatal screening at all. The woman is free to decline this information offer. Not all professionals agreed with this policy, and some argued - contra current policy - that this first question should be skipped, “*because many people actually do not know what it [prenatal screening] entails. How could you say ‘yes’ or ‘no’ to this question when you do not exactly know what this test is for?*” (I3 medical specialist)

In order to reach the aim of prenatal screening, it is of paramount importance that pregnant women or couples can make informed choices for or against a screening offer [15, 17–20]. Informed choice is often defined as “a choice that is based on relevant knowledge, consistent with the decision maker’s values and behaviourally implemented” [34, 35]. This means that women should understand the purpose of the test and its potential risks and implications [36], because they may be confronted with “a large number of further decisions which [they] might have wished to avoid if [they] had been aware of the consequences before screening” [18]. To help women make informed choices pre-test counselling is offered. During pre-test counselling women are presented with information about the purpose, nature, scope and validity [18] and complete information about diseases, including e.g. “name(s) and general characteristics of the major disorder(s),” possible treatments, possible unexpected or unclear findings of the test and kinds of test-outcome [20]. Furthermore, pre-test counselling for first-trimester prenatal screening should be conducted at a designated moment, clearly separated from information provision about other aspects of antenatal care, such as lifestyle, health aspects (e.g. screening for HIV) and birth planning [15].

Many professionals noticed that when women talked about their reasons for choosing prenatal screening, they often mentioned wanting to be reassured about the health of their child. Professionals thought that women sometimes do not realise in advance what kinds of outcomes they might face and difficult choices they might have to make: women *“have to realise that if [they] opt for NIPT and a congenital disorder is found, [they] kind of jump on a train on which [they] might not want to be. [… I] hear people say that they are in a rollercoaster*.” (I8, medical specialist).

Some professionals thought that especially in the case of NIPT this might be a problem. The previous screening programme in the Netherlands was step-wise: the first step was a FCT, which provided only a risk estimate for aneuploidies. Then women had to think carefully about invasive follow-up testing, taking into account the risk of miscarriage. Women could choose whether or not to undergo invasive testing to obtain a diagnostic result. Professionals thought that this step-wise process gradually prepared women for the obtaining of an abnormal test result. They thought that, with NIPT, by contrast, women will opt for an easy test and – in one single step – may be confronted with an almost ‘diagnostic outcome’ at once. NIPT “*gives the idea of a decisive outcome*.” (I10, midwife) As said, this idea is not accurate, as diagnostic follow-up testing is required also with NIPT. The odds that the result turns out false positive, however, are much lower.

In order to protect women from the negative consequences of uninformed choices, professionals emphasised that counselling plays a crucial role. Counselling serves to explain women’s options and to correct misunderstandings of tests and disabilities, but should also explore the norms and values and the attitudes of women towards having a child with a disability. During counselling women should be encouraged to think about their views about testing, about having a child with a disability and termination of pregnancy: *“Yes, I think, that with a few standard questions [the counsellor] will [be able to] achieve a lot. Just to trigger [women], let’s say [to think about the consequences of NIPT]. That does not necessarily take a lot of time. [… As a counsellor, you could ask women:] What does Down syndrome mean to you*?” (I3, medical specialist).

Respondents took the view that the current quality of counselling in the Netherlands is moderate, and needs improvement: professionals should pay more attention to and spend more time on pre-test counselling. Dedicated counselling sessions will help women understand that the aim of prenatal screening for chromosomal abnormalities is different from the aims of antenatal care (i.e. maintaining and/or improving the health of the pregnant woman and the foetus). Some respondents, who were medical specialists, feared that professionals underestimate the importance of (non-directive) counselling for NIPT and should be aware that the ease of the test, requiring only a maternal blood sample, and its high reliability may lead to less informed choices. One study suggested that professionals might indeed attach less importance to informed consent for a non-invasive test compared to an invasive test [37].

To conclude, in order to reach the aim of prenatal screening - reproductive autonomy - informed choice is of crucial importance. Counselling should be non-directive and of high quality, and include deliberation on personal values of women, in order to achieve informed choice and promote this aim.

### Proportionality

In the identified ethical framework, the pillar of proportionality of screening programmes entails balancing benefits and harms, following the original screening criteria for population screening formulated by Wilson and Jungner, complemented with additional criteria from the WHO [15, 19, 38]. To assess benefits and harms, the quality of the test and the test offer, including the laboratory procedures, counselling and education of professionals should be evaluated [15]. According to the ESHG/ASHG and the Dutch Health Council, the benefits and harms or costs depend on the way NIPT will be offered, as a first-tier screening test or second-tier screening test, after FCT [15, 17]. When NIPT would replace FCT as a first-tier test, it might have the benefit of fewer false positive results for trisomy 21, 18, and 13, but on the other hand might also lead to a loss of other findings that can be identified on ultrasound as part of FCT. Experts must decide whether the benefits of a better test performance of NIPT regarding the three trisomies will outweigh this loss of diagnostic yield when the first-trimester ultrasound is removed from the screening programme.

For pregnant women or couples, prenatal screening for foetal abnormalities has the benefit of offering reproductive choices regarding an affected pregnancy, including termination of pregnancy or being able to prepare for the birth of an affected child, relief from anxiety in case of a negative test result and the reduction of invasive follow-up tests [15, 17–20]. Harms for pregnant women are related to false reassurance, burden of decision making and anxiety in case of false-positive outcomes and incidental findings, which can be of unclear clinical significance and might cause needless worries [15]. Respondents held that these harms are inevitable but acceptable. Yet the possibility of incidental findings needs to be explained during pre-test counselling. It has been suggested that making the choice to terminate a desired pregnancy after receiving an abnormal test result may be harmful, as well [20].

According to professionals, women might be faced with unwanted choices they have to make, because they may not have been fully aware of the consequences of prenatal screening beforehand, as some medical specialists indicated in the interviews: *“But sometimes I see people saying: ‘I never would have wanted this choice. This is a horrible choice you’re giving me. I don’t want it. This is a wanted pregnancy. If I had not known that this child has Down syndrome I would go for [continuation of this pregnancy], I am sure. But now I have a choice and I am going to hesitate’.”* (I7, medical specialist).

### Justice

The third pillar of the ethical framework is justice. The principle of justice in prenatal screening relates to equal access to prenatal screening for all pregnant women, to policy questions concerning reimbursement of prenatal screening, and to equal distribution of healthcare resources [15, 17–20]. Equal access to prenatal screening means that differences in personal resources may not cause disparities in access to prenatal screening programmes: women’s choices not to participate in screening should not be based upon a lack of financial resources [15, 17–20]. That would imply that prenatal screening should be offered especially to women with limited financial resources, either free of charge or against a small fee. On the other hand, it could be argued that a (small) payment might serve the aim of reproductive autonomy as it may “increases awareness that there is truly a choice to be made” [15].

Professionals recognised this dilemma concerning the reimbursement of prenatal screening. Respondents mentioned the impact of payment on the uptake of prenatal screening. A midwife suggested that the fee that is currently asked for FCT in the Netherlands (165 euros) has much impact and might explain the large difference in the uptake of FCT as compared to the 20-week ultrasound, which is offered free of charge: “*I am curious, when [NIPT] will be reimbursed and [as a counsellor] you explain to people the possibility of having a NIPT, whether they would say: ‘If it is reimbursed and it gives information about the health of my child, of course I want [to use NIPT].’ They do the same for the 20-week scan. I really wonder whether the difference [in uptake for the FCT and the 20-week ultrasound] is that big because people say: ‘I [do want to] give birth to a child with Down syndrome but not to a child with spina bifida’. I do not believe that [differences in attitudes explain the] difference between 30% [the uptake of the FCT] and 95% [the uptake of the 20-week ultrasound].*” (I10, midwife)

Some professionals suggested that a financial contribution by women might serve as a helpful barrier, making women aware of the importance of the choice. It could prevent women from opting for a test ‘just because it is possible and does not cost any money’, and thus protect them against ill-considered testing. On the other hand, respondents mentioned two objections to payment as a barrier for test uptake. Firstly, professionals thought that some women refrain from screening because of lack of money: “*There are a lot of people for whom [165 euros] is a lot of money that can buy a lot of baby clothes.”* (I13, medical specialist) They thought that lack of money is not a good reason to decline screening. Professionals think that when screening is offered, it should be reimbursed to guarantee unhindered access. Secondly, asking a contribution is in contradiction with equality in healthcare: “*Yes, it is a barrier, but for whom are you creating a barrier? For a specific group of people who cannot pay for it*.” (I3, medical specialist)

Experts should understand that while requiring a personal financial contribution may serve a purpose (i.e. improving informed choice), it may also, and more importantly, create disparities in access to prenatal screening, which is undesirable and contrary to one of the moral pillars of prenatal screening: justice.

### Societal aspects

Self-determination is not only a matter of individual freedom, but also has a societal dimension, and it may be threatened by for example group pressure or societal views about testing [18, 19]. One of the concerns related to group pressure is that it might lead to less-autonomous choices among pregnant women. This would be problematic, it is argued, because the aim of prenatal screening, reproductive autonomy, will not be reached when women fail to make informed choices [15, 17–20].

Furthermore, it is thought that the offer of prenatal screening for chromosomal abnormalities might also imply a discriminatory message to individuals and groups living with specific diseases [15, 17–20]. This objection is known as the ‘disability rights critique’ of prenatal screening and holds that discriminatory messages are inseparable from prenatal screening [15, 17]. This critique may apply both to the sheer societal availability of prenatal screening programmes and to individual women’s choices. In response, it is underlined that the aim of prenatal screening is not preventing the birth of disabled people, but promoting reproductive autonomy [17]. Also, studies have shown that women’s reasons for the selective termination of their pregnancies include prevention of a life of severe suffering and not being able to create the best conditions to care for a child with a disability [17, 39–43], which does not support this critique. However, with the introduction of NIPT, the uptake of first-trimester screening might increase and the number of persons with disabilities might decrease. This is not in itself problematic, but it might become problematic if a low prevalence of disabilities will negatively affect the position of persons with disabilities, and render the option to continue an affected pregnancy less attractive. Therefore the practice of prenatal screening should be evaluated continuously in comparison to its aim [17]. Moreover, a negative perception of people with a disability can be redressed with public information and education [19]. The WHO concludes that just non-discriminatory societal settings are important for making a free choice: “It is important to prevent discrimination and to provide improved support services for individuals and families with genetic conditions. The absence of adequate services for people with hereditary disabilities undermines the principle of free choice for couples at risk of having children with such disabilities” [20].

Professionals did not think that the uptake of prenatal screening would increase dramatically, although they suggested that an easier test is less likely to be declined and might become self-evident. They observed however that the need to participate in prenatal screening is not as self-evident to many pregnant women as the need for other tests in pregnancy. Besides, “*there will always be people who do not want to know [about health risks of their foetus], who just want a care-free pregnancy, and [who feel that] every child is welcome*.” (I13 medical specialist) Moreover, professionals think that women will not choose to terminate pregnancies more often because women who participate in prenatal screening generally have desired pregnancies, and do not wish to undergo termination of pregnancy for trivial reasons [18]. Also, according to respondents, specific cultural aspects in the Netherlands might in part explain the low uptake of NIPT, as compared to other countries. In the Dutch prenatal screening programme, midwives play important roles in pre-test counselling, rather than medical specialists [44]. Among midwives, there is a tendency to avoid medical interference in the pregnancy. Also, in society, a rather positive public image of Down syndrome prevails. Professionals held the opinion that the fear that fewer people with Down syndrome will be born when NIPT is introduced, is not justified. Respondents thought that people with a disability are accepted in the Netherlands and that there is good care available for handicapped people. However, they agreed that care and support should be guaranteed to counteract possible negative consequences of prenatal screening, including discrimination.

In sum, societal aspects and concerns such as an increase in test uptake and a decrease in people born with disabilities are recognized, but disputed in the literature as well as among professionals. However, it is acknowledged that arrangements should be made (i.e. ensuring quality of care for the disabled) to counteract possible negative consequences.

## Discussion

The four pillars of the ethical framework can be used to evaluate the potential expansion of the scope of NIPT. Below, four limits are proposed to the responsible expansion of the scope of NIPT in the future. These limits provide ethical guidance for professionals and policy-makers who are working in the field of NIPT and will be shaping its development and further implementation in the future.

### Limits set by the aim of prenatal screening

In the five documents it is explicitly stated that although the aim of prenatal screening is not to maximise reproductive choice indefinitely, there is room for expansion of the screening offer [15, 17, 20]. In the interviews several professionals indicated that a broader test will contribute to the aim of prenatal screening because an expanded NIPT allows for detecting more disorders than trisomy 21, 18 and 13: “*People do not want a test for Down syndrome, but a test for a healthy child.*” (I15)

However, an expanded scope might affect informed choice as a precondition of reproductive autonomy. When NIPT includes a high number of diseases, it will be difficult in pre-test counselling to discuss all possible test outcomes in detail, “including the full range of variability in the manifestations” of these diseases [15]. Testing for more abnormalities might thus “paradoxically undermine rather than serve or enhance reproductive autonomy.” [15] A clinical professional feared that “*people have no idea what the results [of NIPT] can be and what these could mean to them. I am sure about this, because for Down syndrome it is already the case [that people do not understand what the outcome means to them].”* (I7, medical specialist)

This raises the question how to best inform pregnant women prior to the test. It has been suggested in documents and by some of our respondents that information about the possible outcomes of prenatal screening should be presented as categories of disorders: the scope of NIPT can be narrow or broad, with results pertaining to severe or non-severe disorders and early- or late-onset disorders. When the scope of NIPT expands to such an extent that it becomes impossible to describe in detail all possible test outcomes during pre-test counselling, the counsellor “should describe the general characteristics of the categories of disorders tested for (e.g., mental disability or neurological impairment). Women will receive intensive counselling after a foetal diagnosis.” [20] This model of informed choice is sometimes referred to as ‘generic consent’, which is thought to be a solution for complex counselling and has already been discussed in the context of genetic screening. Generic consent aims to prevent ‘information overload’ and to avoid the provision of information that is “pointless or counterproductive” [45]. The question arises whether generic consent offers enough information to enable people to make a truly informed choices [46, 47]. The ESHG/ASHG and the Dutch Health Council have their reservations about generic consent [15, 17], because “the feasibility of this model has not yet been empirically tested in the prenatal context” and it remains unclear how *informed* generic consent would be [15]. The extent to which generic consent can be informed consent should be studied in line with previous studies on informed choice in the context of prenatal screening. These studies showed highly variable percentages of women having made informed choices: 89%, 77,9%, 51% and 44% [35, 48–50]. Some of that variation might be explained by variation in the nature and the quality of pre-test counselling practices, which will likely affect the ‘informedness’ of women’s choices to a great extent, also in the context of an expanded NIPT. In practice, it is not clear whether a sufficient number of professionals will be available to counsel large numbers of pregnant women and their partners, and whether they will have enough time to explain the details of the test and facilitate informed decision-making. In some countries, measures have been put in place to counter this problem, including the use of decision aids and the additional training of midwives in NIPT counselling [51]. Another solution might be a change in the *focus* of counselling, from technical-medical aspects to women’s values or goals related to screening. As respondents suggested, too, counselling is more than providing information; women should be triggered to think about why they would want prenatal screening and what they would do in case of an abnormal test result, to make them more aware of their attitude towards undergoing prenatal screening. Attitude is defined as the general feeling of ‘favourableness’ or ‘unfavourableness’ for testing [34]. Triggering women to think about testing might lead to a process of deliberation and evaluation of pros and cons, which, according to several authors, should be part and parcel of an informed choice [49]. Professionals could play a role in this deliberation and help women to formulate their values, for instance in accordance with the interpretive model of the physician-patient relationship, as described by Emanuel and Emanuel [52]. This model entails that the healthcare professional helps to elicit the norms and values of a patient.

We would suggest that in this process, the necessary technical information about the test could support or influence the attitude, but is not sufficient or even essential to the quality of decision-making. Shifting the focus of counselling from ‘conveying knowledge about screening’ to ‘exploring women’s attitude towards screening’ might improve women’s and their partners’ decision-making processes, even in the context of an expanded scope of screening and, in combination with decision aids, takes away the time pressure to explain all clinical and technical details of NIPT.

Professionals differed in their opinions about whether women should be given a say in decisions regarding the scope of the screening offer. Some professionals suggested that a list of options should be offered from which women could choose, whereas others believed that experts should determine which (categories of) disorders should be included in the test. The main reason for preferring a predetermined offer was that women might not have the information – or the capacity to understand the information – required to make a decision about the adequate scope of NIPT. Another study of opinions of professionals showed that a majority of respondents preferred a predetermined offer or a fixed list of disorders to be tested [53].

A second category of problems arises with the dual aim of prenatal screening within antenatal care systems [15]. Some routinely offered prenatal screening tests are used to improve pregnancy outcomes or the health condition of the mother or the baby, such as the blood test for rhesus status in RhD-negative women. The rhesus test is currently offered as a separate test but could – for reasons of efficiency – be combined in one test with NIPT for autosomal aneuploidies. An objection to a combination of this test with screening for aneuploidies is the possible confusion in women about what test they should accept or decline, and for what reasons. Prenatal screening for aneuploidies is aimed at reproductive autonomy and requires non-directive counselling [15]. The term ‘non-directiveness’ refers to the absence of coercion or the withholding of advice, in order to respect the autonomy of a patient [54]. According to Ten Have, as cited in Oduncu, non-directiveness means that the expert who provides information about genetic conditions “should not, in any respect, try to influence the decision made by the persons who are counselled or screened. (…) his aim is merely to provide information and to help the patients or clients to work through possible options.” [54] For prevention-aimed screening in antenatal care (e.g. screening for hypertension or rhesus status), it may not be objectionable for health professionals to recommend or insist on participation, because this type of screening promotes the health of the mother and the foetus, but for autonomy-aimed screening, directive counselling is not appropriate [15, 55]. In sum, one (expanded) NIPT that combines two aims and two - opposed - modes of counselling is not desirable.

NIPT is meant to offer reproductive options, but not to screen foetuses for all kinds of medical problems. For instance, children are usually not allowed to undergo predictive testing for (untreatable) late-onset diseases because this might affect their right to an open future and their right not to know unwanted predictive information [15, 17–20]. The principle to defer testing until adulthood applies to unborn children as well. Prenatal screening is not meant as a medical screening of future children: its scope should thus be limited to those conditions for which expecting parents may consider terminating the pregnancy. To protect the unborn child’s right not to know, ‘conditional access’ models have been proposed for women who want information about late-onset diseases: testing for late-onset diseases, including some sex chromosomal aneuploidies, will only be offered if women “expressed the clear intention to choose abortion if a predisposition for a late-onset diseases is found.” [56] However, as termination of a pregnancy is, and should continue to be, the result of a voluntary decision, women who change their minds about an earlier expressed intention cannot be forced to terminate an affected pregnancy. Therefore, it cannot be excluded that children may be born in the knowledge of carrying a mutation for a late-onset disease. Further research should focus on the consequences of living with this information for both parents and children and on its effects on their relationship [18, 19].

NIPT may contribute to the aim of prenatal screening: the promotion of reproductive autonomy. On the basis of the first pillar of the ethical framework for prenatal screening, however, limits can be set to the morally responsible expansion of the scope of NIPT: NIPT should generate only test outcomes that are relevant to reproductive decision-making, and informed choice should be (made) possible through adequate pre-test counselling.

### Limits set by proportionality

The expansion of the scope of NIPT also raises questions concerning proportionality. According to the Dutch Health Council, proportionality is an important requirement of prenatal screening, and benefits of each ‘test’ (for each condition) to be included in the screening offer should outweigh the harms [17]. Professionals noted that it may be beneficial to include more disorders in a test because that means that more reproductive choices can be made: *“There are children who are born with a severe disorder. Then we do an exome analysis to see what the cause is. Then we find, say, in 40% of the cases, a new mutation, in a crucial gene, which the parents do not have. In the future it may be possible to detect that [mutation] in maternal blood*.” (I15, lab specialist) Several professionals gave the example of the 22q11 deletion, which is associated with a severe phenotype. Studies on the attitudes of pregnant women towards an expanded scope of prenatal screening showed that women thought that it may be valuable especially to include severe disorders with no or short life expectancy in a screening test [57, 58]. Women also wanted to learn about sex chromosomal aneuploidies [59, 60] and about specific other aneuploidies, but were hesitant about learning about conditions with unknown or variable phenotypic expression. They were uncertain about what the benefit would be of knowing about such conditions [59].

Proportionality concerns might limit the expansion of the scope, on at least three points. Firstly, when genome-wide analyses are used in NIPT, it might be difficult to assess the clinical validity of many among the huge number of abnormalities that can be detected. Offering a test for disorders without knowing the validity might lead to false positives and false negatives, cause harm to pregnant women, and challenge the proportionality of including the disorders [17]. Professionals mentioned that outcomes should be actionable for pregnant women. When tests are not reliable (i.e. clinically valid), they provide few actionable options. Moreover, uncertain test outcomes might lead to unnecessary anxiety or insecurity in pregnant women, which is objectionable: *“I think that, when you introduce uncertainty in the pregnancy, it will become difficult. If you [can say that you] are sure that the child is disabled, then this is understandable for people, and they will be able to prepare [for the birth of a disabled child] or to decide that they do not want this. But if you say, ‘we actually do not know what it means exactly; (…) it can turn out better than expected, but the child can also turn out severely disabled.’ Well, what should you do, as parents*?” (I3, medical specialist) Several other professionals stated that in practice this should not pose a big problem, as only a small number of abnormalities that are currently being detected in labs are of unknown or little-known clinical validity. These will need to be discussed between expecting parents and clinical geneticists specialised in prenatal diagnosis.

A second point that several respondents stressed is that NIPT has shortcomings: NIPT is not a diagnostic test, and it still requires invasive follow-up. An expanded scope might lead to an increasing number of positive test results for a wide range of disorders, which will include false positive results that need confirmation by (unnecessary) invasive diagnostic testing. This is problematic, because a reduction of invasive tests as compared to FCT is seen as one of the important benefits of NIPT [15, 18, 19].

A third point that might limit the scope of NIPT is the burden of the decision to terminate a pregnancy. Some disorders may not be sufficiently severe to justify their inclusion in the NIPT; they may not meet the first screening criterion of Wilson and Jungner: “The condition sought should be an important health problem.” [61] However, professionals mentioned that it is hard to define what ‘serious’ or ‘non-serious’ diseases are. In the documents it is stated, for instance, that severity should not be determined at all: “It would be dangerous to create medical, legal, or social definitions of ‘serious’, because these could infringe on couples’ lives in several ways.” [20] Expecting parents are the ones who should indicate whether they consider a disorder to be serious or not, in their life situation [20]. Although it will be difficult in practice to draw the lines, the seriousness of disorders can serve as an (arguable) limit to the expanding scope of NIPT.

From the pillar of proportionality a few additional limits can be derived for the expansion of the scope of NIPT: in order for tests to be included in an expanded scope of NIPT, they should be clinically valid. Especially the positive predictive value should be high, as confirmatory testing through invasive procedures will still be required and is associated with risks, costs and burdens. NIPT should not be offered for trivial conditions.

### Limits set by justice aspects

When using the ethical framework to evaluate an expansion of the scope NIPT, the pillar of justice is less prominent than the other three pillars. However, there are three issues that arise from the pillar of justice. Firstly, when NIPT is offered as an expanded test, it should be available equally for every pregnant woman [17]. Equal access to healthcare is considered to be a fundamental right that should preclude the exclusion of specific groups from healthcare services [62]. Women should not face restrictions to having reproductive options. Ideally, all women should have access to the same information about their foetus, and the scope of first-trimester prenatal screening should be equal for all women. When expanded NIPT is made available only to women who have an increased risk of trisomy 21, 18 or 13 as a second-tier test after FTC, for instance, low-risk pregnant women will not have access to information about the foetus other than the three more common trisomies detected through FCT, whereas high-risk women will [17]. For this reason, justice would require making NIPT available as a first-tier test to all women (or restricting the scope of NIPT as a second-tier test). Also, it is important to note that diagnostic follow-up testing should be made available to women who have undergone NIPT, in line with the criterion of Wilson and Jungner that in screening programmes, diagnostic follow-up testing should be available to those found to be at risk [38]. This is of special importance in countries in which access to follow-up testing is not self-evident.

A second aspect, according to the International Bioethics Committee, is that education is a matter of justice: “Persons with a lower education level and lower health literacy are denied the information which is required to exercise their freedom and autonomy.” [19] Some women may not be able to understand all relevant information pertaining to the screening offer, which is necessary to make an informed choice. The expansion of NIPT will only exacerbate this inequality [19], it is feared, as the test becomes more elaborate and more complex, and decision-making places higher demands on women’s health literacy.

A third concern is that an expanded NIPT could challenge a justifiable distribution of healthcare resources. As resources are scarce and should be distributed equally, efforts must be taken to demarcate the scope of prenatal screening tests to prevent unnecessary follow-up of clinically insignificant findings. Besides, when prenatal screening is offered within the context of a public health programme and is upheld by taxpayers, there should be transparency with regard to the utility of the test [15]. This also underlines the importance of ensuring the proportionality of a test.

When considering the costs of prenatal screening it should be noted that a widespread implementation and uptake of prenatal screening programmes is likely to lead to the birth of fewer affected children, which reduces the costs associated with their healthcare and support. Although this should not be an aim of prenatal screening, these long-term costs savings are undeniably part of a cost-effectiveness analysis of new screening tests [15].

From the pillar of justice another limitation can be derived: expanded NIPT should be available for all pregnant women, which may increase the costs of the programme. This limitation may change over time as the technology improves and becomes cheaper.

### Limits set by societal aspects

In discussions on the expansion of the scope of NIPT, concerns are reiterated that have already been raised in the context of earlier prenatal screening programmes, such as discrimination and stigmatisation of people with chronic diseases. New societal aspects, unique to expanded NIPT, are raised as well. Professionals noted in the interviews, for instance, that a benefit of an expanded scope could be a removal of the focus of prenatal testing on Down syndrome. Down syndrome is the most common of the three trisomies and in the Netherlands first-trimester screening for chromosomal abnormalities is often referred to as a ‘test for Down syndrome’. By expanding the scope of prenatal screening this focus could shift, which might reduce concerns related to discriminatory messages conveyed by the screening programme. This benefit of the expansion is also acknowledged by parents of children with Down syndrome, who experience the focus on Down as stigmatising for their children [63]. On the other hand, the Dutch Health Council mentioned that expanded NIPT is not free from the allegation of stigmatisation either, as, for instance, a list of selected disorders can be thought of as ‘subjective’ and vulnerable to stigmatisation of specific groups, too [17]. According to some professionals, an expanded scope might reduce the acceptance of children with a disability: “*With 22q11 deletion, [children] can be mentally retarded, etc. When people hear a story like that, they tend to terminate [the pregnancy]. I find it very hard. Everybody wants a healthy child; I understand that. So it is good to have these options. On the other hand, I am afraid that, when more [screening] becomes possible, what space is there for children with a disability? I find it terrible that there may be no respect or no care [for these children].”* (I10, midwife).

Adverse societal consequences of an expanded scope are also mentioned by pregnant women and parents of children with Down syndrome, who fear a loss of diversity in society and a ‘slippery slope’, implying that people might want to start testing for increasingly trivial abnormalities [57, 63]. However, respondents questioned whether these consequences of an expanded scope will occur and denied that society will eventually be without children with a disorder or disability. Although it is difficult to predict the societal consequences (if any) of NIPT or how these would limit the expansion of its scope, it is clear that negative consequences for people with disabilities should be mitigated, and the practice of prenatal screening should be monitored continuously, not only with a focus on the risks and benefits for individuals, but also for its wider societal implications.

## Conclusion

An expansion of the scope of NIPT fits the aim of prenatal screening, as it contributes to more reproductive options for pregnant women and couples. However, drawing on the broadly shared ethical framework for prenatal screening as well as on the findings of our qualitative study of professionals’ opinions and experiences of the translation of the pillars of this framework in practice, we conclude that expansion of the scope of NIPT is not unlimited. Four moral limits can be set to demarcate a responsible expansion of the scope of NIPT. Firstly, informed choice as a central precondition for prenatal screening should limit its scope: when NIPT is expanded to include more chromosomal or sub-microscopic abnormalities, and relevant pre-test information about the test becomes more elaborate and more complex, counsellors will need to improve pre-test counselling to uphold its quality. This requires new models for counselling, with a special focus on generic information about possible test outcomes and on expecting parents’ attitudes and values in relation to prenatal screening. Secondly, any expansion of NIPT should be proportionate: the test should be clinically valid and useful to women. Findings that generate mainly anxiety and for which no courses of action are available, do not meet the criterion of proportionality. Thirdly, respect for the right of the future child to an open future excludes testing for late-onset disorders when women or couples know beforehand that they will not terminate the pregnancy based on the results. Finally, healthcare resources should be justly distributed: when possible, NIPT should be made available to all pregnant women either free of charge or for a small sum. At the same time, any expansion of the scope of NIPT should be based upon a favourable assessment of the benefits of including additional ‘tests’ for additional disorders in proportion to the costs and burdens. Both in the literature and in our interview study of professionals’ opinions, we observed differences in the sense of urgency or importance that is attributed to each of the four limitations. We contend that the criterion of reproductive autonomy as the aim of prenatal screening as well as that of proportionality – or a positive balance between the benefits and burdens for pregnant women and their future children – should together be guiding in decisions whether particular disorders should be tested or communicated to women or couples. This means that for example, depending on the test performance, disorders that are comparable to trisomies 13, 18 and 21 in terms of severity could be included in the NIPT. Over the next decade, those working in the field of NIPT may strive to maximise the potential benefits of NIPT and include more abnormalities in the screening test, keeping these moral limits to a justified scope of NIPT in mind.

## Abbreviations

ASHG: American Society of Human Genetics
ESHG: European Society of Human Genetics
FCT: First trimester combined test
IBC: UNESCO International Bioethics Committee
NIPT: Non-Invasive Prenatal Test
TRIDENT-1: Trial by Dutch laboratories for Evaluation of Non-Invasive Prenatal Testing
WHO: World Health Organisation
